# The risk of clinical complications and death among pregnant women with COVID-19 in the Cerner COVID-19 cohort: a retrospective analysis

**DOI:** 10.1186/s12884-021-03772-y

**Published:** 2021-04-16

**Authors:** Fares Qeadan, Nana A. Mensah, Benjamin Tingey, Joseph B. Stanford

**Affiliations:** Department of Family and Preventive Medicine, University of Utah School of Medicine, 375 Chipeta Way, Ste A, Salt Lake City, UT 84108 USA

## Abstract

**Background:**

Pregnant women are potentially a high-risk population during infectious disease outbreaks such as COVID-19, because of physiologic immune suppression in pregnancy. However, data on the morbidity and mortality of COVID-19 among pregnant women, compared to nonpregnant women, are sparse and inconclusive. We sought to assess the impact of pregnancy on COVID-19 associated morbidity and mortality, with particular attention to the impact of pre-existing comorbidity.

**Methods:**

We used retrospective data from January through June 2020 on female patients aged 18–44 years old utilizing the Cerner COVID-19 de-identified cohort. We used mixed-effects logistic and exponential regression models to evaluate the risk of hospitalization, maximum hospital length of stay (LOS), moderate ventilation, invasive ventilation, and death for pregnant women while adjusting for age, race/ethnicity, insurance, Elixhauser AHRQ weighted Comorbidity Index, diabetes history, medication, and accounting for clustering of results in similar zip-code regions.

**Results:**

Out of 22,493 female patients with associated COVID-19, 7.2% (*n* = 1609) were pregnant. Crude results indicate that pregnant women, compared to non-pregnant women, had higher rates of hospitalization (60.5% vs. 17.0%, *P* < 0.001), higher mean maximum LOS (0.15 day vs. 0.08 day, *P* < 0.001) among those who stayed < 1 day, lower mean maximum LOS (2.55 days vs. 3.32 days, *P* < 0.001) among those who stayed ≥1 day, and higher moderate ventilation use (1.7% vs. 0.7%, *P* < 0.001) but showed no significant differences in rates of invasive ventilation or death. After adjusting for potentially confounding variables, pregnant women, compared to non-pregnant women, saw higher odds in hospitalization (aOR: 12.26; 95% CI (10.69, 14.06)), moderate ventilation (aOR: 2.35; 95% CI (1.48, 3.74)), higher maximum LOS among those who stayed < 1 day, and lower maximum LOS among those who stayed ≥1 day. No significant associations were found with invasive ventilation or death. For moderate ventilation, differences were seen among age and race/ethnicity groups.

**Conclusions:**

Among women with COVID-19 disease, pregnancy confers substantial additional risk of morbidity, but no difference in mortality. Knowing these variabilities in the risk is essential to inform decision-makers and guide clinical recommendations for the management of COVID-19 in pregnant women.

**Supplementary Information:**

The online version contains supplementary material available at 10.1186/s12884-021-03772-y.

## Introduction

The COVID-19 pandemic continues to wreak havoc in the United States and around the world. As of February,16, 2021, the cumulative case-counts in the US had surpassed twenty-seven million [1]. As researchers and experts alike look towards mitigation strategies, there is a critical need to investigate how the pandemic impacts specific subgroups. One critical population of interest is pregnant women. Pregnancy imposes physiological and immunological changes that have important implications on the severity and outcomes of viral conditions [2, 3]. Although data for COVID-19 pregnant women are still emerging, prior epidemiologic evidence suggests that many viral infections may result in a higher risk of fatalities and clinical complications among pregnant women [4, 5]. During the 2004 SARS pandemic, higher rates of complications and death were reported among pregnant women compared to non-pregnant women [6, 7]. Similar adverse outcomes were observed for pregnant women during the 2009 H1N1 influenza pandemic [8]. Data on MERS, albeit limited, also points to increased morbidity during pregnancy [9]. Accordingly, the Centers for Disease Control and Prevention (CDC) have emphasized the need for pregnant women to take rigorous precautionary measures against COVID-19 [10].

While some researchers suggest a reason for an increased risk of adverse outcomes for viral infections is the presence of a pregnancy-induced immune-suppressed state, others propose that the physiologic and immune responses during pregnancy in some instances may be protective rather than suppressive [11, 12]. Thus far, most studies on COVID-19 infections among pregnant women have been limited by small sample sizes, or have been focused on specific pregnancy outcomes such as miscarriage or stillbirths [13–15]. Larger national studies of COVID-19 in pregnancy have been reported, but with limitations of substantial missing data on pre-existing chronic conditions and demographic variables [16]. Our study aims to fill this gap by assessing the impact of pregnancy on adverse outcomes of COVID-19 using national data from the Cerner COVID-19 De-Identified Data cohort. We aim to evaluate the adjusted risk of the following COVID-19 complications in relation to pregnancy: hospitalization, the maximum length of hospital stays, moderate ventilation, invasive ventilation, and death. As a secondary aim, we assessed the impact across a range of pre-existing co-morbidity.

## Methods

### Settings

We used data from the *Cerner COVID-19 De-Identified Data Cohort,* which provides patient-level information on diagnoses, laboratory tests, procedures, medication, and encounter information over the patient-visit continuum. Cerner obtains its data from participating hospitals as described in detail as follows. *“Cerner Real-World Data is extracted from the EMR of hospitals in which Cerner has a data use agreement. Encounters may include pharmacy, clinical and microbiology laboratory, admission, and billing information from affiliated patient care locations. All admissions, medication orders and dispensing, laboratory orders and specimens are date and time stamped, providing a temporal relationship between treatment patterns and clinical information. Cerner Corporation has established Health Insurance Portability and Accountability Act-compliant operating policies to establish deidentification for Cerner Real-World Data.”* [17, 18]

Cerner extracts its data from the electronic health record systems (EHRs) of participating US hospitals—for the COVID-19 De-Identified Data Cohort, data from 62 health systems were used. It then subjects the data to rigorous cleaning and standardization, including the use of a multipoint-match algorithm to identify and remove duplicate records. Records are also standardized across disparate EHR coding systems and fully de-identified. The most recent data refresh, used for this analysis, was performed in June 2020. The records of patients identified as having an encounter associated with a 1) diagnosis code of possible exposure or infection of COVID-19 or 2) positive lab result for COVID-19 testing (several types of tests were performed but confidentiality of the tests cannot be revealed within the manuscript) were included in the data set. These were referred to as the COVID-19 “associated” cohort. A more specific sub-cohort was restricted only to those patients that had an encounter associated with a 1) diagnosis code of COVID-19 infection or 2) a recent positive lab result (up to two weeks prior) of COVID-19 infection. These were referred to as the COVID-19 “positive” cohort. This cohort was provided to compare with the results of the larger COVID-19 “associated” cohort in supplemental analyses. To provide further insight into these patients’ health histories, available medical information since January 1, 2015, was also included. Of all valid patients from both cohorts, only female patients of adult reproductive age (18–44 years old) were included for analysis. The University of Utah Institutional Review Board has determined that this study does not meet the definitions of Human Subjects Research for using secondary data with no intervention or interaction with an individual, and for having no identifiable private information in the data. Thus, requirements of the informed consent for this study and ethical approval for this study were waived by the University of Utah Institutional Review Board (IRB #136696). All methods were performed in accordance with the relevant guidelines and regulations​.

### Measurements

The outcomes of interest involved 5 indications of clinical complications in COVID-19 associated patients: hospitalization, maximum hospital length of stay (LOS), moderate ventilation, invasive ventilation, and death. Maximum LOS was created by calculating the difference in days between the start and end dates of each patient encounter and taking the maximum difference per patient. Maximum LOS was split further into two sub-groups: 1) those staying less than one day and 2) those staying one day or greater. Hospitalization was a binary indication (yes/no) and flagged “yes” for those patients that had a max LOS of one day or greater. Ventilation was a binary indication (yes/no) of whether a patient ever had a diagnosis, procedure, or encounter result indication that included reliance on either a 1) moderate ventilator (i.e., CPAP or BiPAP) or 2) invasive ventilator (i.e., intubation or tracheostomy). Death was a binary indication (yes/no) of whether a patient died at discharge or any time thereafter during the time of data collection (through June, 2020).

The primary predictor of interest was pregnancy status. This was a binary indicator (yes/no) of whether a patient had any ICD-9/10 diagnosis code of pregnancy no more than 7 and a half months before a qualifying COVID-19 “associated” encounter. This time frame ensured that the pregnancy was generally timely with the COVID-19 “associated” encounter.

The key clinical factor for our secondary aim was patient comorbidity, which was expressed via the Elixhauser comorbidity index (ECI) [19]. This index is analogous to the Charlson comorbidity index (CCI) [20], in that it measures patient comorbidity by calculating a risk-of-death score from each qualifying condition a given patient may have. The scores, from each condition, are then summed and weighted to provide a total score of mortality risk for the patient. Conditions for the ECI are defined by ICD-10 diagnosis codes. The ECI, however, uses a slightly different set of pre-existing conditions as well as a different weighting algorithm. The weighting of this score is performed using the Agency for Health Care Research and Quality (AHRQ) weighting methodology [21]. The ECI provides categorization of scores of less than 0, 0, 1–4, and 5 or higher [22] [23].

Demographic characteristics considered as potential confounders included age in years, race and ethnicity (non-Hispanic (NH) American Indian/Alaskan Native (AI/AN), NH Asian/Pacific Islander (API), NH Black/African American (Black), NH White, NH other/mixed/unknown race, Hispanic or Latino), insurance status (private, government/miscellaneous, Medicaid, Medicare, self-pay, missing), and 1-digit zip-code region. 1-digit zip-codes were grouped into four regions (northeast, southeast, midwest, west) for descriptive presentation of the data, but were left in their original form for inferential modeling. Additional clinical factors considered as potential confounders were binary (yes/no) indications of COVID-19 medication use: Hydroxychloroquine, Remdesivir, Decadron and Prednisone, Aspirin and Plavix, and anticoagulants. These were also compiled from Multum medication records. An additional clinical factor was a binary indication of whether the patient had a history (defined by ICD 9/10 diagnosis code) of gestational diabetes (DM).

### Statistical analysis

Overall demographic and clinical characteristics were presented for female patients, 18–44 years old, in the COVID-19 “associated” cohort and were stratified by pregnancy status. Categorical variables were presented with frequencies and percentages. Continuous variables were presented with medians and interquartile ranges (Q1-Q3) because they were not normally distributed. Categorical variables with sufficient sample sizes were compared using a chi-square test and small samples were compared using Fisher’s exact test. Nonparametric continuous variables were compared using a Wilcoxon rank-sum test. For an initial presentation of pregnancy status on clinical complications in COVID-19 “associate” women, the 5 outcomes were stratified by those pregnant and those not pregnant. The percentages of hospitalization, ventilation, and death were compared using a Chi-squared test. Median maximum LOS, for each sub-group, was compared using a Wilcoxon rank-sum test.

The adjusted association of pregnancy with clinical complications was assessed by fitting mixed-effects regression models. All models involved a random effect of 1-digit zip-code to account for clustering of results in similar zip-code regions. Outcomes of hospitalization, moderate/invasive ventilation, and death were fit with logistic regression models. An exponential regression model was fit for each sub-group of maximum LOS, because maximum LOS followed a continuous, exponential distribution. All models were fit with an indication of pregnancy status as the primary exposure while adjusting for ECI, history of gestational DM, age, race/ethnicity, insurance, and COVID-19 medication-use. Adjusted odds ratios (ORs) with 95% confidence intervals (CIs) were reported for the logistic models, and adjusted exponentiated coefficients relating to the percentage change in expected maximum LOS with 95% CIs were reported for the exponential model. For logistic regression models, an area (AUC) under the receiver operating characteristic curve (ROC) was calculated to assess the models’ ability to correctly classify outcome categories. For the exponential model, the coefficient of determination (R^2^) was calculated to estimate the percentage of variation in max LOS as explained by the model predictors.

Scatterplot figures were constructed to visually represent the impact of comorbidity and pregnancy on the predicted clinical complication outcomes. Each figure showed the predicted outcome against the ECI. In addition, different lines were fit for those pregnant and those not pregnant during COVID-19 “associated” encounters. Smoothed lines were fit amongst the data by generalized additive regression (GAM) models with shrinkage cubic-regression splines.

Sensitivity analyses were performed by stratifying all previous regression models by demographic and clinical characteristics of interest: categorized age (18–34 years old and 35–44 years old), race and ethnicity, insurance, ECI, and DM history (yes or no). In addition, all previously mentioned statistical analyses were repeated for women 18–44 years old from the COVID-19 “positive” cohort. Results pertaining only to these patients were compared against the results of the full COVID-19 “associated” cohort. Finally, a sub-analysis was performed in which postpartum women from the COVID-19 associated cohort were identified and the four outcomes of interest were presented among this group. Outcomes in postpartum women were compared with the original outcomes in pregnant women. All hypothesis tests were two-sided with a significance level of 5%. R version 3.6.1 (R Foundation for Statistical Computing, Vienna, Austria) was used to perform all analyses. In addition, R package “comorbidity” (version 0.5.3) was used to calculate comorbidity scores [24].

## Results

Overall, there were 22,493 total female patients, 18–44 years old, from the COVID-19 “associated” cohort. The median (Q1-Q3) age was 31 [25–38] and the majority of patients were Hispanic/Latino (41.0% (9232)) with 26.1% (5872) being NH White and 17.6% (3966) being NH Black. The majority of patients used private insurance (48.9% (11,010)) and 23.5% (5285) used Medicaid. Most patients came from the southeast United States (45.1% (10,134)). Pregnant patients (compared to non-pregnant patients) were younger (median age: 29 vs. 32), had a lower distribution of Hispanic/Latino ethnicity (34.8% vs. 41.5%), had a higher distribution of Medicaid use (36.0% vs. 22.5%) and lower distribution of self-pay (7.5% vs. 14.2%), and lower distribution from the southeast United States (39.3% vs. 45.5%). All *P* < 0.001 (Table 1). When looking at history of chronic diseases, non-pregnant women generally had higher percentages of all disease types. However, pregnant patients had a significantly higher percentage reporting obesity than non-pregnant patients (20.6% (331) vs. 17.1% (3567, *P* < 0.001). Pregnant patients had lower use reporting of all COVID-19 related medications than non-pregnant patients, except for anticoagulants (13.6% vs. 8.2%, *P* < 0.001). Pregnant patients also reported higher rates of gestational DM history than non-pregnant women (10.1% vs. 2.0%, *P* < 0.001) (Table 2).

**Table 1:** **Demographic characteristics of COVID-19 associated female patients (18–44 years old) by pregnancy status**

Characteristic	Total n(%^2^)	Pregnant^1^ n(%^2^)	Not Pregnant n(%^2^)	p-value^6^
Total	22,493 (100)	1609 (7.2^3^)	20,884 (92.8^3^)	
Age (Years)^4^	31 (25–38)	29 (25–33)	32 (25–38)	**< 0.001**^**7**^
Age (Years) Categorized				**< 0.001**
18–24	4913 (21.8)	389 (24.2)	4524 (21.7)	
25–34	9128 (40.6)	910 (56.6)	8218 (39.4)	
35–44	8452 (37.6)	310 (19.3)	8142 (39.0)	
Race and Ethnicity				**< 0.001**
Non-Hispanic American Indian or Alaska Native	517 (2.3)	14 (0.9)	503 (2.4)	
Non-Hispanic Asian or Pacific Islander	527 (2.3)	40 (2.5)	487 (2.3)	
Non-Hispanic Black or African American	3966 (17.6)	285 (17.7)	3681 (17.6)	
Non-Hispanic White	5872 (26.1)	446 (27.7)	5426 (26.0)	
Non-Hispanic Other^5^	2379 (10.6)	264 (16.4)	2115 (10.1)	
Hispanic or Latino	9232 (41.0)	560 (34.8)	8672 (41.5)	
Insurance				**< 0.001**
Private	11,010 (48.9)	708 (44.0)	10,302 (49.3)	
Government/Misc	819 (3.6)	31 (1.9)	788 (3.8)	
Medicaid	5285 (23.5)	580 (36.0)	4705 (22.5)	
Medicare	430 (1.9)	13 (0.8)	417 (2.0)	
Self-Pay	3083 (13.7)	121 (7.5)	2962 (14.2)	
Missing	1866 (8.3)	156 (9.7)	1710 (8.2)	
Region^8^				**< 0.001**
Northeast	3493 (15.5)	451 (28.0)	3042 (14.6)	
Southeast	10,134 (45.1)	633 (39.3)	9501 (45.5)	
Midwest	2969 (13.2)	195 (12.1)	2774 (13.3)	
West	5283 (23.5)	184 (11.4)	5099 (24.4)	
Missing	614 (2.7)	38 (2.4)	468 (2.2)	

^1^ Defined by ICD-9 and ICD-10 codes

^2^% = column percentage

^3^% = out of total (22,493) percentage

^4^ median (Q1-Q3)

^5^ other or unknown

^6^ Chi-Square Test (unless otherwise noted)

^7^ Wilcoxon-Rank Sum Test

^8^ Northeast: 0 (Connecticut, Massachusetts, Maine, New Hampshire, New Jersey, Rhode Island, Vermont), 1 (Delaware, New York, Pennsylvania); Southeast: 2 (DC, Maryland, North Carolina, South Carolina, Virginia, West Virginia), 3 (Alabama, Florida, Georgia, Mississippi, Tennessee); Midwest: 4 (Indiana, Kentucky, Michigan, Ohio), 5 (Iowa, Minnesota, Montana, North Dakota, South Dakota, Wisconsin), 6 (Illinois, Kansas, Missouri, Nebraska), 7 (Arkansas, Louisiana, Oklahoma, Texas), West: 8 (Arizona, Colorado, Idaho, New Mexico, Nevada, Utah, Wyoming), 9 (Alaska, California, Hawaii, Oregon, Washington)

**Table 2:** Clinical characteristics of COVID-19 associated female patients (18–44 years old) by pregnancy status

Characteristic	Pregnant^1^ n(%^2^)	Not Pregnant n(%^2^)	p-value^4^
Total	1609	20,884	
History of chronic diseases^7^			
Congestive heart failure	9 (0.6)	359 (1.7)	**< 0.001**^**5**^
Cardiac arrhythmias	167 (10.4)	2224 (10.7)	0.74
Valvular disease	22 (1.4)	299 (1.4)	0.91
Pulmonary circulation disorders	12 (0.7)	344 (1.7)	**0.01**
Peripheral vascular disorders	2 (0.1)	248 (1.2)	**< 0.001**^**5**^
Hypertension, uncomplicated	104 (6.5)	2355 (11.3)	**< 0.001**
Hypertension, complicated	7 (0.4)	412 (2.0)	**< 0.001**^**5**^
Paralysis	0 (0.0)	141 (0.7)	**< 0.001**^**5**^
Other neurological disorders	35 (2.2)	904 (4.3)	**< 0.001**
Chronic pulmonary disease	203 (12.6)	3640 (17.5)	**< 0.001**
Diabetes, uncomplicated	48 (3.0)	1392 (6.7)	**< 0.001**
Diabetes, complicated	17 (1.1)	766 (3.7)	**< 0.001**
Hypothyroidism	81 (5.0)	1042 (5.0)	> 0.99
Renal failure	2 (0.1)	353 (1.7)	**< 0.001**^**5**^
Liver disease	26 (1.6)	948 (4.6)	**< 0.001**
Peptic ulcer disease	3 (0.2)	138 (0.7)	**0.01**^**5**^
AIDS/HIV	4 (0.2)	72 (0.3)	0.66^5^
Lymphoma	0 (0.0)	49 (0.2)	**0.048**^**5**^
Metastatic cancer	1 (0.1)	82 (0.4)	**0.03**^**5**^
Solid tumor without metastasis	9 (0.6)	244 (1.2)	**0.03**
Rheumatoid arthritis/collagen vascular diseases	16 (1.0)	459 (2.2)	**0.002**
Coagulopathy	71 (4.4)	636 (3.1)	**0.003**
Obesity	331 (20.6)	3567 (17.1)	**< 0.001**
Weight loss	14 (0.9)	391 (1.9)	**0.01**
Fluid and electrolyte disorders	265 (16.5)	3313 (15.9)	0.58
Blood loss anemia	15 (0.9)	196 (0.9)	> 0.99
Deficiency anemia	88 (5.5)	1021 (4.9)	0.34
Alcohol abuse	15 (0.9)	502 (2.4)	**< 0.001**
Drug abuse	85 (5.3)	1176 (5.6)	0.58
Psychoses	14 (0.9)	304 (1.5)	*0.07*
Depression	42 (2.6)	734 (3.5)	*0.06*
Elixhauser AHRQ weighted comorbidity (continuous score)	0 (0–0)^3^	0 (0–3)^3^	**0.001**^**6**^
Elixhauser AHRQ weighted comorbidity (categorical score)			**< 0.001**
< 0	386 (24.0)	4166 (19.9)	
0	822 (51.1)	11,031 (52.8)	
1–4	116 (7.2)	2237 (10.7)	
> =5	285 (17.7)	3450 (16.5)	
COVID-19 Medications			
Hydroxychloroquine	38 (2.4)	525 (2.5)	0.77
Remdesivir	3 (0.2)	39 (0.2)	> 0.99
Decadron or Prednisone	36 (2.2)	1029 (4.9)	**< 0.001**
Aspirin and Plavix	0 (0.0)	22 (0.1)	0.40
Other anticoagulant	219 (13.6)	1721 (8.2)	**< 0.001**
History of Gestational Diabetes	163 (10.1)	411 (2.0)	**< 0.001**

^1^ Defined by ICD-9 and ICD-10 codes; ^2^% = column percentage; ^3^ median (Q1-Q3)

^4^ Chi-square test (unless otherwise noted); ^5^ Fisher’s Exact test

^6^ Wilcoxon Rank-Sum Test; ^7^ Up till January 1, 2015 (these are the diseases that make up the Elixhauser comorbidity index);

Table 3 shows the comparisons in complications between pregnant and non-pregnant patients. Pregnant women, compared to non-pregnant women, had higher rates of hospitalization (60.5% vs. 17.0%, *P* < 0.001), higher maximum LOS among those who stayed less than one day in the hospital(0.15 days vs. 0.08 days, *P* < 0.001), and higher moderate ventilation use (1.7% vs. 0.7%, *P* < 0.001). Among those who stayed one day or greater in the hospital, pregnant women showed significantly lower max LOS than non-pregnant women (2.55 days vs. 3.32 days, *P* < 0.001). No significant differences were found in invasive ventilation or death.

**Table 3:** Complications in COVID-19 associated pregnant and non-pregnant women (18–44 years old)

Outcome	Pregnant n(%^1^)	Not Pregnant n(%^1^)	p-value^5^
Hospitalized	**974 (60.5)**	**3546 (17.0)**	**< 0.001**
Maximum length of hospital stay (Less than one day)^2^	**0.15 (0.08–0.23)**	**0.08 (0.05–0.14)**	**< 0.001**^**6**^
Maximum length of hospital stay (One day or greater)^2^	**2.55 (2.02–3.43)**	**3.32 (2.05–6.59)**	**< 0.001**^**6**^
Moderate Ventilation^3^	**28 (1.7)**	**149 (0.7)**	**< 0.001**
Invasive Ventilation^4^	26 (1.6)	396 (1.9)	0.48
Deceased	4 (0.2)	100 (0.5)	0.26

^1^% = column percentage

^2^ Days, median (Q1-Q3)

^3^ Less-invasive ventilator indications like CPAP or BIPAP machines

^4^ More severe and invasive ventilator indications, including tracheostomy

^5^ Chi-squared test (unless otherwise noted)

^6^ Wilcoxon Rank Sum Test

**Fig. 1:**
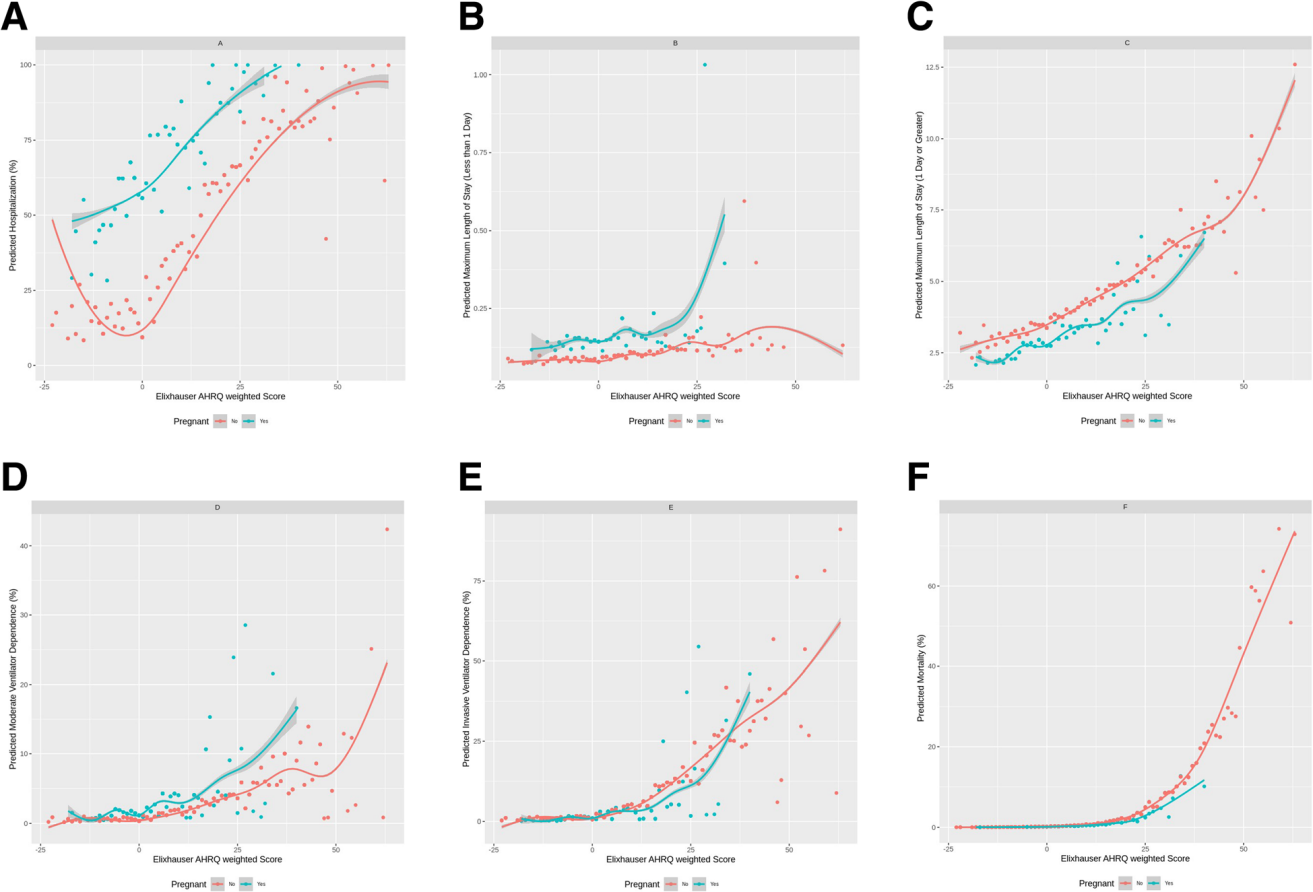
**a-f** Predicted outcomes (hospitalization, maximum length of stay (< 1 day, > = 1 day), moderate ventilation, invasive ventilation, and death) vs. Elixhauser AHRQ weighted score, among COVID-19 associated female patients (18–44 years old) by pregnancy status

The adjusted associations with the 5 clinical complications are reported in Table 4. Pregnant women, compared to non-pregnant women, saw higher odds in hospitalization (aOR: 12.26; 95% CI (10.69, 14.06)), moderate ventilation (aOR: 2.35; 95% CI (1.48, 3.74)), and higher max LOS among those staying less than one day in the hosptial (${e}^{\hat{\beta}}$: 1.78; 95% CI (1.67, 1.90)). Among those staying one day or more in the hospital, pregnant women had significantly lower max LOS than non-pregnant women (${e}^{\hat{\beta}}$: 0.90; 95% CI (0.85, 0.95)). No significant associations were found with invasive ventilation or death (Table 4). Figures 1A-F report the predicted outcomes by comorbidity scores (ECI) and grouped by pregnant and non-pregnant status. In all cases, as ECI increases so too does the predicted clinical complication. However, pregnant patients (compared to non-pregnant patients) have higher average predicted complications for hospitalization, max LOS (< 1 Day), and moderate ventilation.

**Table 4:** Adjusted association of pregnancy and other clinical and demographic variables, with hospitalization, maximum length of hospital stay, moderate ventilation, invasive ventilation, and death, among COVID-19 associated female patients (18–44 years old)

Variables	Hospitalization	Max LOS (< 1 Day)	Max LOS (> = 1 Day)	Moderate Ventilation	Invasive Ventilation	Death
	aOR^1^ (95% CI)	${e}^{\hat{\beta}}$ ^2^ (95% CI)	${e}^{\hat{\beta}}$ ^2^ (95% CI)	aOR^1^ (95% CI)	aOR^1^ (95% CI)	aOR^1^ (95% CI)
Pregnant						
No	1 [Reference]	1 [Reference]	1 [Reference]	1 [Reference]	1 [Reference]	1 [Reference]
Yes	**12.26 (10.69, 14.06)**	**1.78 (1.67, 1.90)**	**0.90 (0.85, 0.95)**	**2.35 (1.48, 3.74)**	0.89 (0.57, 1.40)	0.83 (0.30, 2.33)
Age (years) [5 years increment]^3^	**1.07 (1.03, 1.10)**	**1.03 (1.02, 1.04)**	**1.04 (1.02, 1.06)**	1.07 (0.96, 1.19)	**1.19 (1.10, 1.28)**	**1.21 (1.05, 1.40)**
Race and Ethnicity						
Non-Hispanic White	1 [Reference]	1 [Reference]	1 [Reference]	1 [Reference]	1 [Reference]	1 [Reference]
Non-Hispanic American Indian or Alaska Native	**1.43 (1.05, 1.94)**	**0.84 (0.77, 0.93)**	**1.47 (1.27, 1.70)**	2.19 (0.99, 4.86)	**3.83 (2.19, 6.70)**	**2.76 (1.12, 6.77)**
Non-Hispanic Asian or Pacific Islander	1.21 (0.93, 1.59)	1.02 (0.94, 1.11)	**1.21 (1.06, 1.38)**	0.75 (0.23, 2.48)	1.22 (0.65, 2.28)	1.58 (0.53, 4.73)
Non-Hispanic Black or African American	0.91 (0.79, 1.04)	**1.10 (1.06, 1.14)**	1.07 (1.00, 1.14)	1.08 (0.69, 1.68)	0.79 (0.58, 1.08)	1.12 (0.63, 1.99)
Non-Hispanic Other	1.09 (0.93, 1.28)	**1.05 (1.02, 1.09)**	1.01 (0.95, 1.07)	1.26 (0.75, 2.13)	0.86 (0.58, 1.27)	1.84 (0.93, 3.66)
Hispanic or Latino	**0.85 (0.75, 0.96)**	0.99 (0.94, 1.03)	1.07 (1.00, 1.16)	0.92 (0.59, 1.42)	0.70 (0.51, 0.95)	0.83 (0.47, 1.48)
Insurance						
Private	1 [Reference]	1 [Reference]	1 [Reference]	1 [Reference]	1 [Reference]	1 [Reference]
Government/Misc	0.82 (0.64, 1.06)	**0.91 (0.85, 0.97)**	0.97 (0.85, 1.11)	1.46 (0.61, 3.53)	1.06 (0.55, 2.05)	**1.98 (1.16, 3.40)**
Medicaid	**1.46 (1.30, 1.64)**	**1.15 (1.11, 1.19)**	**1.13 (1.07, 1.19)**	1.36 (0.91, 2.03)	1.21 (0.91, 1.61)	2.01 (0.88, 4.60)
Medicare	**1.43 (1.06, 1.93)**	**1.29 (1.17, 1.43)**	1.03 (0.92, 1.17)	1.03 (0.46, 2.28)	0.91 (0.54, 1.52)	1.39 (0.62, 3.11)
Self-Pay	**0.79 (0.67, 0.93)**	0.98 (0.95, 1.02)	0.98 (0.90, 1.07)	1.45 (0.86, 2.47)	0.88 (0.57, 1.36)	1.39 (0.62, 3.11)
Missing	**2.00 (1.71, 2.33)**	1.01 (0.96, 1.07)	**1.19 (1.11, 1.27)**	**1.74 (1.05, 2.90)**	**2.10 (1.51, 2.93)**	**2.17 (1.16, 4.05)**
Elixhauser AHRQ weighted Comorbidity Score [10 units increment]^4^	**1.96 (1.84, 2.08)**	**1.12 (1.09, 1.14)**	**1.16 (1.13, 1.18)**	**1.37 (1.19, 1.57)**	**1.38 (1.32, 1.44)**	**3.15 (2.76, 3.61)**
Gestational Diabetes						
No	1 [Reference]	1 [Reference]	1 [Reference]	1 [Reference]	1 [Reference]	1 [Reference]
Yes	**1.58 (1.24, 2.01)**	0.98 (0.90, 1.07)	0.96 (0.87, 1.06)	0.86 (0.38, 1.94)	0.79 (0.41, 1.53)	–
Decadron and Prednisone						
No	1 [Reference]	1 [Reference]	1 [Reference]	1 [Reference]	1 [Reference]	1 [Reference]
Yes	**2.15 (1.78, 2.59)**	**1.28 (1.20, 1.36)**	**1.26 (1.17, 1.35)**	**2.49 (1.68, 3.69)**	**2.34 (1.77, 3.11)**	–
Anticoagulant						
No	1 [Reference]	1 [Reference]	1 [Reference]	1 [Reference]	1 [Reference]	1 [Reference]
Yes	**72.73 (61.15, 86.50)**	**3.67 (3.27, 4.12)**	**1.36 (1.30, 1.43)**	**12.11 (8.53, 17.18)**	**13.76 (10.82, 17.50)**	–
AUC	0.89	–	–	0.88	0.93	0.90
R^2^	–	0.12	0.15	–	–	–

^1^ Adjusted odds ratio from mixed-effect logistic regression model (clustering on one-digit zip-code)

^2^ adjusted exponentiated coefficients (mixed-effect exponential regression model clustering on one-digit zip-code) relating to percentage change in expected maximum length of hospital stay

^3^ Change in odds (or change in % of response for exponential model) for each 5 unit increase in predictor

^4^Change in odds (or change in % of response for exponential model) for each 10 unit increase in predictor

The results of the stratified analyses reveal generally similar findings between all demographic/clinical groups for hospitalization, maximum LOS, invasive ventilation, and death (Table 5). Increased outcomes for pregnant patients (compared to non-pregnant) were seen across all groups for hospitalization and maximum LOS (< 1 Day). Among those staying a day or greater in the hospital, pregnant women showed lower max LOS than non-pregnant women. This was significant among patients 18–34 years old, whites, AIAN, Medicaid holders, and patients without a diabetes history. No significant associations were seen among invasive ventilation and death. For moderate ventilation, differences were seen among age groups and race/ethnicity groups. Among patients 35–44, pregnant women saw significantly higher odds of moderate ventilation compared to non pregnant women (aOR: 3.74; 95% CI (1.65, 8.52)). No such difference was detected among patients 18–34. NH Black and NH other race patients, that were pregnant, saw significantly higher odds of moderate ventilation than those that were not pregnant (aOR: 4.51; 95% CI (1.96, 10.39), and aOR: 6.40; 95% CI (2.68, 15.28) respectively). Again no such difference was seen among white and Hispanic/Latino patients. Additionally, when restricted to patients from the COVID-19 “positive” cohort (*n* = 8806; Pregnant *n* = 748, Non-pregnant *n* = 8058), the results are similar to that of the COVID-19 “associated” cohort (see Supplemental Tables A-C and Supplemental Figs. 1A-E).

**Table 5:** Stratified sensitivity analyses of the adjusted association of pregnancy with hospitalization, maximum length of hospital stay, moderate ventilation, invasive ventilation, and death, among COVID-19 associated female patients (18–44 years old)

Variables	Hospitalization	Max LOS (< 1 Day)	Max LOS (> = 1 Day)	Moderate Ventilation	Invasive Ventilation	Death
	aOR (95% CI)	${e}^{\hat{\beta}}$ (95% CI)	${e}^{\hat{\beta}}$ (95% CI)	aOR (95% CI)	aOR (95% CI)	aOR (95% CI)
Age (Years)						
18–34	**12.79 (10.94, 14.94)**^**1**^	**1.74 (1.62, 1.87)**	**0.92 (0.86, 0.98)**	1.06 (0.24, 4.66)	0.93 (0.53, 1.63)	0.65 (0.20, 2.18)
35–44	**11.71 (8.63, 15.88)**	**1.90 (1.63, 2.22)**	0.93 (0.82, 1.06)	**3.74 (1.65, 8.52)**	0.93 (0.43, 2.03)	1.10 (0.15, 8.28)
Race and Ethnicity						
Non-Hispanic White	**12.64 (9.79, 16.32)**	**1.89 (1.63, 2.20)**	**0.89 (0.80, 0.99)**	1.62 (0.60, 4.37)	1.17 (0.52, 2.61)	0.75 (0.10, 5.77)
Non-Hispanic American Indian or Alaska Native	**13.15 (3.46, 50.02)**	1.75 (0.74, 4.15)	**0.49 (0.28, 0.84)**	-^2^	–	8.08 (0.56, 115.45)
Non-Hispanic Asian or Pacific Islander	**12.58 (5.14, 30.76)**	**1.71 (1.01, 2.89)**	0.76 (0.54, 1.06)	–	–	–
Non-Hispanic Black or African American	**9.85 (7.12, 13.63)**	**1.55 (1.35, 1.79)**	0.89 (0.77, 1.03)	**4.51 (1.96, 10.39)**	1.54 (0.64, 3.72)	1.46 (0.18, 12.07)
Non-Hispanic Other	**15.14 (10.08, 22.75)**	**2.22 (1.81, 2.74)**	0.91 (0.81, 1.03)	**6.40 (2.68, 15.28)**	1.19 (0.45, 3.13)	0.59 (0.07, 4.79)
Hispanic or Latino	**11.77 (9.33, 14.86)**	**1.72 (1.57, 1.89)**	0.97 (0.87, 1.07)	1.63 (0.55, 4.78)	1.04 (0.43, 2.48)	–
Insurance						
Private	**16.23 (13.12, 20.08)**	**1.79 (1.64, 1.96)**	0.96 (0.88, 1.05)	**3.81 (1.89, 7.66)**	1.18 (0.58, 2.40)	0.59 (0.07, 4.70)
Government/Misc	**8.05 (3.31, 19.58)**	1.40 (0.91, 2.17)	1.02 (0.69, 1.51)	–	–	–
Medicaid	**9.75 (7.79, 12.22)**	**1.75 (1.55, 1.97)**	**0.88 (0.80, 0.97)**	**2.87 (1.40, 5.88)**	1.52 (0.80, 2.89)	1.38 (0.39, 4.82)
Medicare	3.16 (0.84, 11.83)	1.30 (0.68, 2.46)	2.00 (0.92, 4.35)	–	–	–
Self-Pay	**10.05 (6.14, 16.47)**	**1.93 (1.58, 2.35)**	1.03 (0.84, 1.27)	**5.55 (1.54, 20.00)**	–	–
Missing	**11.64 (6.93, 19.54)**	**1.82 (1.31, 2.51)**	**0.82 (0.71, 0.96)**	0.58 (0.07, 4.47)	0.86 (0.30, 2.46)	–
Elixhauser AHRQ weighted Comorbidity Index						
< 0	**10.96 (8.29, 14.50)**	**1.80 (1.54, 2.11)**	**0.87 (0.78, 0.96)**	**3.41 (1.42, 8.17)**	0.96 (0.33, 2.80)	**–**
0	**24.46 (19.93, 30.02)**	**1.79 (1.65, 1.94)**	0.95 (0.88, 1.03)	**3.41 (1.06, 10.98)**	1.05 (0.31, 3.54)	**–**
1–4	**9.10 (5.57, 14.87)**	**1.55 (1.25, 1.92)**	0.93 (0.74, 1.16)	**3.67 (1.02, 13.18)**	0.98 (0.23, 4.21)	**–**
> =5	**3.15 (2.29, 4.34)**	**1.68 (1.39, 2.03)**	0.94 (0.82, 1.07)	**2.00 (1.07, 3.75)**	1.03 (0.59, 1.80)	1.07 (0.38, 3.07)
Diabetes History						
No	**12.12 (10.55, 13.92)**	**1.78 (1.67, 1.90)**	**0.88 (0.83, 0.93)**	**3.14 (1.92, 5.14)**	1.03 (0.64, 1.67)	1.27 (0.45, 3.61)
Yes	**10.81 (4.66, 25.07)**	**2.30 (1.24, 4.26)**	0.97 (0.73, 1.29)	–	0.59 (0.14, 2.58)	–

^1^ Odds/percentage change of **column outcome** for those pregnant compared to those not among **row group,** adjusted for all other predictors in Table 3 for sufficient sample sizes, COVID-19 medications and disease history (upon need) removed for insufficient sample sizes;

^2^ “-“=Insufficient sample size

The results of the sub-analysis (Table 6), looking at outcomes in postpartum women from the COVID-19 associated cohort, show higher complications for these women compared to pregnant women from the same cohort. When compared to pregnant women, postpartum women had higher percentages of hospitalization (90.5% vs. 60.5%, *P* < 0.001), higher max LOS less than one day (0.20 days vs. 0.15 days, *P* = 0.09), and higher invasive ventilation (5.3% vs. 1.6%, *P* = 0.003).

**Table 6:** Complications in COVID-19 associated non-pregnant, pregnant, and postpartum women (18–44 years old)

Outcome	Not Pregnant n(%^1^)	Pregnant n(%^1^)	Postpartum n(%^1^)	p-value^5^
Total	**20,884**	**1609**	**190**	
Hospitalized	3546 (17.0)	974 (60.5)	172 (90.5)	< 0.001^6^
Maximum length of hospital stay (Less than one day)^2^	0.08 (0.05–0.14)	0.15 (0.08–0.23)	0.20 (0.14–0.32)	0.09^7^
Maximum length of hospital stay (One day or greater)^2^	3.32 (2.05–6.59)	2.55 (2.02–3.43)	2.63 (2.11–3.88)	0.19^7^
Moderate Ventilation^3^	149 (0.7)	28 (1.7)	4 (2.1)	0.77
Invasive Ventilation^4^	396 (1.9)	26 (1.6)	10 (5.3)	0.003
Deceased	100 (0.5)	4 (0.2)	0 (0.0)	> 0.99

^1^% = column percentage

^2^ Days, median (Q1-Q3)

^3^ Less-invasive ventilator indications like CPAP or BIPAP machines

^4^ More severe and invasive ventilator indications, including tracheostomy

^5^ Comparison of pregnant with postpartum women, Fisher’s exact test unless otherwise noted

^6^ Chi-Square test

^7^ Wilcoxon rank-sum test

## Discussion

Our study investigated the extent to which pregnancy imposes the risk of severe COVID-19 complications among COVID-19 associated patients and COVID-19 positive patients, and examined the association across the range of prior comorbidity. We focused on five principal outcomes: maximum length of hospital stays, moderate ventilation, invasive ventilation, and death. In the COVID-19 associated group, we found significant differences in COVID-19 outcomes among COVID-19 associated pregnant women, and non-pregnant COVID-19 associated women. Notably, our findings showed that COVID-19 associated pregnant women were more than 12 times as likely to be hospitalized, more than twice as likely to require moderate ventilation and had a maximum LOS of less than 1 Day two times greater than that of COVID-19 associated non-pregnant. Perhaps most importantly, we did not observe an increased risk for invasive ventilation or increased risk of death among pregnant COVID-19 associated women. We observed similar results after stratifying by age and other demographic or clinical characteristics. We further restricted our analysis to confirmed COVID-19 positive patients and found comparable results. In the COVID-19 confirmed group, pregnant COVID-19 pregnant women were more than ten times as likely to be hospitalized, nearly three times as likely to require moderate ventilation and had a maximum LOS of less than 1 Day almost two times greater that of COVID-19 confirmed non-pregnant women.

Our findings parallel results from CDC’s initial report on SARS-CoV − 2 infection among pregnant women [16] yet contradicts certain findings from CDC’s updated report [25] on COVID-19 and pregnancy. For instance, similar to our results, CDC’s previous report showed that although pregnancy was associated with a heightened risk of hospitalization, intensive care admission, and mechanical ventilator receipt, pregnancy was not linked to increased risk of death among reproductive aged women with confirmed COVID-19 illness [16]. However, CDC’s newest study on COVID-19 and pregnancy reported that pregnant COVID-19 diagnosed women were nearly twice as likely to die compared to non-pregnant COVID-19 diagnosed women [25]. Similarly, researchers observed a case fatality rate 13.6 times higher among pregnant COVID-19 patients in Washington State [26]. In comparison to the above mentioned studies, our research is strengthened by a more complete assessment of demographic variables, clinical characteristics, and prior comorbidities. However, the overall low rate of deaths (only 4 deaths) among pregnant women in our sample may have hindered the ability to capture accurate mortality.

The current recommended standards of care for pregnant COVID-19 patients might explain our observed results. For instance, the CDC, the American College of Obstetricians and Gynecologists (ACOG), and the Society for Maternal-Fetal Medicine (SMFM) urge special considerations for the management of COVID-19 during pregnancy [27–29]. Examples of these guidelines include hospitalization of pregnant women diagnosed with COVID-19 in facilities with maternal and fetal monitoring capabilities when warranted and the use of multispecialty team based approach during treatment [30]. We argue that because of these recommendations and the limited scientific knowledge on COVID-19 and pregnancy, providers are likely taking precautionary measures in caring for their pregnant COVID-19 patients. Therefore, the fact that pregnancy was linked to increased hospitalization, higher LOS of less than 1 Day, moderate ventilation but not invasive ventilation or death lends additional support to the notion that the observed results may in part reflect provider behavior or current clinical recommendations. The additional attention provided for pregnant women may reduce their risk of severe morbidity or death, but this cannot be definitively identified in these observational data. Further studies are needed to examine the contribution of provider behavior on COVID-19 health outcomes for pregnant women. Even so, the effects of specific physiologic changes during pregnancy such as decreased lung capacity, reduced cell-mediated immunity, and increased heart rate on the outcome of viral infections such as SARS-COV-2 infections cannot be overlooked [31, 32].

Other clinical factors associated with adverse COVID-19 outcomes were increasing comorbidity index score and being on Medicare or Medicaid. Several recent COVID-19 studies have reported a link between increasing comorbidities with increasing COVID-19 complications [33–35]. Similarly, being on Medicare or Medicaid has also been associated with poor COVID-19 outcomes [36]. In particular, Medicaid can be used as a proxy for lower socioeconomic status as Medicaid eligibility is based on federal poverty level guidelines [37]. Medicare beneficiaries who are under the age of 65 such as those present in our study may have disabilities or severe medical conditions such as end-stage renal disease [38]. Taken together, lower socioeconomic status and underlying medical conditions have critical implications on worsening COVID-19 outcomes [33, 39–41].

Concerning racial and ethnic differences, we found that the risk of hospitalization and higher LOS of < 1 Day among pregnant patients, compared to those who are not pregnant, to be significant and comparable across race/ethnicity groups (aOR ranges from 11.77–15.14 and ${e}^{\hat{\beta}}$ ranges from 1.55–2.22 across races) (Table 5). However; NH Black and NH other race pregnant patients had significantly higher odds of moderate ventilation than those that were not pregnant; a finding that was not statistically significant among white and Hispanic/Latino patients. Current literature has uncovered the disproportionate overall burden of COVID-19 on minority populations, including Blacks and Native Americans [42, 43]. In a Brazillian study, researchers reported a nearly two-fold increase in maternal mortality due to COVID-19 for Black women [44]. Several reasons, including higher prevalence of underlying conditions, disproportionate representation in essential worker occupations, housing, and living conditions, and structural racism, have been identified as reasons for poor outcomes observed among minority populations [44–47]. When focusing on pregnant women only, racial disparities in pregnancy complications persist among women of color when compared to whites with some exceptions for hispanic women which is consistent with our observation. For example, an investigation of racial disparity in pregnancy outcomes at a tertiary care medical center found that while black women were more likely, compared to whites, to remain in the hospital for > 4 days, have higher rates of preterm birth, small-for-gestational age infants, preeclampsia, and stillbirths, Hispanic women were found to have lower odds for preterm birth and when compared to black women, Hispanic women were less likely to experience any adverse pregnancy events, with the exception of gestational diabetes mellitus [48]. Here, we refer to the Hispanic paradox; the positive health outcomes observed among Hispanic populations despite risks associated with lower SES [49] as a likely explanation while acknowledging that other COVID-19 studies have reported adverse effects in Hispanic populations [50] in general but not among pregnant women in particular.

Finally, our stratified sensitivity analysis indicated a nearly fourfold increased risk for moderate ventilation among COVID-19 associated pregnant women aged 35 to 44 years. However, the risk of moderate ventilation among pregnant women aged 18 to 24 years did not yield significant results. In comparison, CDC’s newest study reported a heightened risk of invasive ventilation among COVID-19 pregnant women of all ages, with the highest risk observed among older pregnant women aged 35–44 years old [25]. Given that advanced maternal age has been associated with a host of adverse pregnancy outcomes [51] our findings highlight the need for providers to pay close attention to pregnant women of advanced ages who contract COVID-19.

Our analysis has several limitations. First, our data were limited to only participating health systems, and may not fully represent the general population. Second, our study only included individuals who sought medical care for COVID-19 and may under-represent medically underserved minority populations such as Hispanics/Latinos and NH Blacks who may not seek treatment due to lack of health insurance or other barriers. Thirdly, data on the indication for the intensive care unit (ICU) admission were not available and thus hospitalization may have occurred in other areas outside the ICU. Further, all cases and many of our outcomes and predictors were identified using ICD and LOINC EHR codes, thereby limiting the study’s specificity. Moreover, data from patient EHR are subject to ICD coding errors, may not include all relevant patient diagnoses, and may only capture primary patient complaints, resulting in lower estimates of vital patient health history components. Of note, our low estimates of maternal deaths in comparison to other studies might be partly attributable to possible data entry discrepancies between participating health systems and inconsistencies in data processing and restructuring. Lastly, our primary analysis did not include postpartum women, and given that the postpartum period may constitute a period of greater risks of maternal death [52], their exclusion in our study might underestimate maternal deaths in comparison to studies that include the postpartum period [26, 44]. To address this issue, we have added a subanalysis that compares complications in COVID-19 between postpartum women and pregnant women. Despite these limitations, our study has several strengths. We utilized a large sample size (8000–22,000), sufficient to capture important trends, and conduct informative stratified analyses for a wide variety of characteristics. Our large sample size also allowed for analyses of rare patient populations (i.e. pregnant women with COVID-19) that may be incredibly difficult to identify using alternative means. We also utilized a national database that allowed for an analysis of a diverse and more representative patient population. Finally, to reduce the effects of ICD coding limitations, we applied other methods such as text matching to capture every possible indication in the data.

## Conclusion

Our study indicates that pregnancy confers additional risk with COVID-19 disease for medical complications, but may not ultimately increase mortality in the setting of sufficient medical care. Our results support current recommendations for particular attention and care to pregnant women who incur SARS-COV2 infection and highlight the importance of access to care for these women. Additionally, our findings provide further justification for the CDC and ACOG’s current recommendations [53, 54] regarding the vaccination of pregnant women who are part of recommended priority groups.

## Supplementary Information

**Additional file 1 Table S1.** Demographic characteristics of COVID-19 positive female patients (18–44 years old) by pregnancy status. **Table S2.** Clinical characteristics of COVID-19 positive female patients (18–44 years old) by pregnancy status. **Table B.** Comparisons in complications between COVID-19 positive pregnant and non-pregnant women (18–44 years old). **Table C.** Adjusted variable associations with complications (hospitalization, maximum length of hospital stay, moderate ventilation, invasive ventilation) among COVID-19 positive female patients (18–44 years old). **Fig. S1A-E.** Predicted outcomes (hospitalization, maximum length of stay (< 1 day, > = 1 day), moderate ventilation, and invasive ventilation) vs. Elixhauser AHRQ weighted score, among COVID-19 positive female patients (18–44 years old) by pregnancy status.

## Data Availability

The datasets generated during and/or analyzed during the current study are not publicly available due to restrictions by Cerner; the owner of the data. Data could be accessed by signing a data sharing agreement with Cerner and covering any costs that may be involved.

## Abbreviations

LOS: hospital length of stay
CDC: Centers for Disease Control and Prevention
EHRs: electronic health record systems
ECI: Elixhauser comorbidity index
CCI: Charlson comorbidity index
AHRQ: Agency for Health Care Research and Quality
NH: non-Hispanic
AI/AN: American Indian/Alaskan Native
API: Asian/Pacific Islander
DM: gestational diabetes
ORs: odds ratios
aOR: adjusted OR
CIs: confidence intervals
ROC: receiver operating characteristic
AUC: area under the ROC curve
R2: coefficient of determination
GAM: generalized additive regression
ACOG: American College of Obstetricians and Gynecologists
SMFM: Society for Maternal-Fetal Medicine
