# Supplementary material for: The risk of clinical complications and death among pregnant women with COVID-19 in the Cerner COVID-19 cohort: a retrospective analysis
Source: BMC Pregnancy Childbirth. 2021 Apr 16;21:305. doi: 10.1186/s12884-021-03772-y (PMC8051832; doi:10.1186/s12884-021-03772-y)
Supplement: Supplementary file 1 — Additional file 1 Table S1. Demographic characteristics of COVID-19 positive female patients (18–44 years old) by pregnancy status. Table S2. Clinical characteristics of COVID-19 positive female patients (18–44 years old) by pregnancy status. Table B. Comparisons in complications between COVID-19 positive pregnant and non-pregnant women (18–44 years old). Table C. Adjusted variable associations with complications (hospitalization, maximum length of hospital stay, moderate ventilation, invasive ventilation) among COVID-19 positive female patients (18–44 years old). Fig. S1A-E. Predicted outcomes (hospitalization, maximum length of stay (< 1 day, > = 1 day), moderate ventilation, and invasive ventilation) vs. Elixhauser AHRQ weighted score, among COVID-19 positive female patients (18–44 years old) by pregnancy status. [file 12884_2021_3772_MOESM1_ESM.docx]

**The risk of clinical complications and death among pregnant women with COVID-19**

Fares Qeadan^a^; Nana A. Mensah^a^, Benjamin Tingey^a^; Joseph B. Stanford^a^

*^a^ University of Utah School of Medicine, Department of Family and Preventive Medicine, United States*

Corresponding author:

Fares Qeadan, PhD, MS

Assistant Professor

Department of Family and Preventive Medicine, University of Utah

375 Chipeta Way, Ste A, Room 108 South

Salt Lake City, UT 84108

Phone: (801) 587-3285 (office)

Email: [fares.qeadan@utah.edu](mailto:fares.qeadan@utah.edu)

**Supplemental Tables and Figures**

**Supplemental Table A.1:** Demographic characteristics of COVID-19 positive female patients (18-44 years old) by pregnancy status

| Characteristic | Total  n(%^2^) | Pregnant^1^  n(%^2^) | Not Pregnant  n(%^2^) | | p-value^6^ |
| --- | --- | --- | --- | --- | --- |
| Total | 8,806 | 748 (8.5^3^) | | 8,058 (91.5^3^) |  |
| Age (Years)^4^ | 32 (26-38) | 29 (25-33) | | 33 (26-39) | **<0.001^7^** |
| Age (Years) Categorized |  |  | |  | **<0.001** |
| 18-24 | 1,700 (19.3) | 184 (24.6) | | 1,516 (18.8) |  |
| 25-34 | 3,534 (40.1) | 415 (55.5) | | 3,119 (38.7) |  |
| 35-44 | 3,572 (40.6) | 149 (19.9) | | 3,423 (42.5) |  |
| Race and Ethnicity |  |  | |  | **<0.001** |
| Non-Hispanic Black or African American | 1,883 (21.4) | 158 (21.1) | | 1,725 (21.4) |  |
| Non-Hispanic White | 1,650 (18.7) | 134 (17.9) | | 1,516 (18.8) |  |
| Non-Hispanic Other^5^ | 1,540 (17.5) | 170 (22.7) | | 1,370 (17.0) |  |
| Hispanic or Latino | 3,733 (42.4) | 286 (38.2) | | 3,447 (42.8) |  |
| Insurance |  |  | |  | **<0.001** |
| Private | 3,647 (41.4) | 273 (36.5) | | 3,374 (41.9) |  |
| Medicaid or Medicare | 2,626 (29.8) | 300 (40.1) | | 2,326 (28.9) |  |
| Self-Pay | 1,208 (13.7) | 61 ( 8.2) | | 1,147 (14.2) |  |
| Other^8^ | 1,325 (15.0) | 114 (15.2) | | 1,211 (15.0) |  |
| Region^9^ |  |  | |  | **<0.001** |
| Northeast | 1,740 (19.8) | 219 (29.3) | | 1,521 (18.9) |  |
| Southeast | 3,365 (38.2) | 313 (41.8) | | 3,052 (37.9) |  |
| Midwest | 1,225 (13.9) | 93 (12.4) | | 1,132 (14.0) |  |
| West | 2,205 (25.0) | 73 ( 9.8) | | 2,132 (26.5) |  |
| Missing | 271 ( 3.1) | 50 ( 6.7) | | 221 ( 2.7) |  |

^1^ Defined by ICD-9 and ICD-10 codes

^2^ %=column percentage

^3^ % =row percentage

^4^ median (Q1-Q3)

^5^ Non-Hispanic American Indian or Alaska Native, Non-Hispanic Asian or Pacific Islander, Non-Hispanic other or unknown race

^6^ Chi-Square Test (unless otherwise noted)

^7^ Wilcoxon-Rank Sum Test

^8^ Government/Misc, other, or missing

^9^ Northeast: 0 (Connecticut, Massachusetts, Maine, New Hampshire, New Jersey, Rhode Island, Vermont), 1 (Delaware, New York, Pennsylvania); Southeast: 2 (DC, Maryland, North Carolina, South Carolina, Virginia, West Virginia), 3 (Alabama, Florida, Georgia, Mississippi, Tennessee); Midwest: 4 (Indiana, Kentucky, Michigan, Ohio), 5 (Iowa, Minnesota, Montana, North Dakota, South Dakota, Wisconsin), 6 (Illinois, Kansas, Missouri, Nebraska), 7 (Arkansas, Louisiana, Oklahoma, Texas), West: 8 (Arizona, Colorado, Idaho, New Mexico, Nevada, Utah, Wyoming), 9 (Alaska, California, Hawaii, Oregon, Washington)

**Supplemental Table A.2:** Clinical characteristics of COVID-19 positive female patients (18-44 years old) by pregnancy status

| Characteristic | Pregnant^1^  n(%^2^) | Not Pregnant  n(%^2^) | | p-value^4^ |
| --- | --- | --- | --- | --- |
| Total | 748 | | 8,058 |  |
| History of chronic diseases^3^ |  | |  |  |
| Congestive heart failure | 2 ( 0.3)^2^ | | 136 ( 1.7)^2^ | **0.001^5^** |
| Cardiac arrhythmias | 84 (11.2) | | 907 (11.3) | 0.98 |
| Valvular disease | 6 ( 0.8) | | 103 ( 1.3) | 0.33 |
| Pulmonary circulation disorders | 8 ( 1.1) | | 148 ( 1.8) | 0.16 |
| Peripheral vascular disorders | 0 ( 0.0) | | 92 ( 1.1) | **<0.001^5^** |
| Hypertension, uncomplicated | 33 ( 4.4) | | 1,007 (12.6) | **<0.001** |
| Hypertension, complicated | 2 ( 0.3) | | 162 ( 2.0) | **<0.001^5^** |
| Paralysis | 0 ( 0.0) | | 49 ( 0.6) | **0.02^5^** |
| Other neurological disorders | 6 ( 0.8) | | 353 ( 4.4) | **<0.001** |
| Chronic pulmonary disease | 91 (12.2) | | 1,380 (17.2) | **<0.001** |
| Diabetes, uncomplicated | 23 ( 3.1) | | 743 ( 9.3) | **<0.001** |
| Diabetes, complicated | 8 ( 1.1) | | 419 ( 5.2) | **<0.001** |
| Hypothyroidism | 27 ( 3.6) | | 385 ( 4.8) | 0.16 |
| Renal failure | 1 ( 0.1) | | 165 ( 2.1) | **<0.001^5^** |
| Liver disease | 9 ( 1.2) | | 394 ( 4.9) | **<0.001** |
| Peptic ulcer disease | 0 ( 0.0) | | 46 ( 0.6) | **0.03^5^** |
| AIDS/HIV | 2 ( 0.3) | | 34 ( 0.4) | 0.77^5^ |
| Lymphoma | 0 ( 0.0) | | 25 ( 0.3) | 0.27^5^ |
| Metastatic cancer | 0 ( 0.0) | | 25 ( 0.3) | 0.27^5^ |
| Solid tumor without metastasis | 1 ( 0.1) | | 80 ( 1.0) | **0.01^5^** |
| Rheumatoid arthritis/collagen vascular diseases | 12 ( 1.6) | | 162 ( 2.0) | 0.52 |
| Coagulopathy | 35 ( 4.7) | | 282 ( 3.5) | 0.13 |
| Obesity | 187 (25.0) | | 1,701 (21.2) | **0.02** |
| Weight loss | 3 ( 0.4) | | 146 ( 1.8) | **0.002^5^** |
| Fluid and electrolyte disorders | 138 (18.4) | | 1,490 (18.6) | 0.95 |
| Blood loss anemia | 5 ( 0.7) | | 78 ( 1.0) | 0.55^5^ |
| Deficiency anemia | 32 ( 4.3) | | 441 ( 5.5) | 0.18 |
| Alcohol abuse | 4 ( 0.5) | | 153 ( 1.9) | **0.004^5^** |
| Drug abuse | 29 ( 3.9) | | 358 ( 4.5) | 0.51 |
| Psychoses | 5 ( 0.7) | | 102 ( 1.3) | 0.52^5^ |
| Depression | 21 ( 2.8) | | 249 ( 3.1) | 0.73 |
| Elixhauser AHRQ weighted comorbidity index |  | |  | **<0.001** |
| <0 | 198 (26.5) | | 1,661 (20.6) |  |
| 0 | 355 (47.5) | | 4,045 (50.2) |  |
| 1-4 | 46 ( 6.1) | | 823 (10.2) |  |
| >=5 | 149 (19.9) | | 1,529 (19.0) |  |
| COVID-19 Medications |  | |  |  |
| H[ydroxychloroquine](https://www.google.com/search?rlz=1C1GCEU_enUS890US890&q=Hydroxychloroquine&spell=1&sa=X&ved=2ahUKEwj4toq_t9DrAhUTU80KHWX-CfgQkeECKAB6BAgSECc) | 33 ( 4.4) | | 456 ( 5.7) | 0.18 |
| Remdesivir | 3 ( 0.4) | | 39 ( 0.5) | >0.99 |
| Decadron or Prednisone | 18 ( 2.4) | | 439 ( 5.4) | **<0.001** |
| Aspirin and Plavix | 0 ( 0.0) | | 5 ( 0.1) | >0.99 |
| Anticoagulant | 147 (19.7) | | 1,115 (13.8) | **<0.001** |
| History of Gestational Diabetes | 77 (10.3) | | 200 ( 2.5) | **<0.001** |

^1^ Defined by ICD-9 and ICD-10 codes

^2^ %=column percentage

^3^ Up till January 1, 2015 (these are the diseases that make up the Elixhauser comorbidity index);

^4^ Chi-square test (unless otherwise noted)

^5^Fisher’s Exact test

**Supplemental Table B:** Comparisons in complications between COVID-19 positive pregnant and non-pregnant women (18-44 years old)

| Outcome | Pregnant  n(%^1^) | Not Pregnant  n(%^1^) | p-value^5^ |
| --- | --- | --- | --- |
| Hospitalized | **488 (65.2)** | **2,031 (25.2)** | **<0.001** |
| Maximum length of hospital stay (Less than one day)^2^ | **0.18 (0.11-0.27)** | **0.10 (0.05-0.16)** | **<0.001^6^** |
| Maximum length of hospital stay (One day or greater)^2^ | **2.49 (1.99-3.58)** | **4.01 (2.35-7.39)** | **<0.001^6^** |
| Moderate Ventilation ^3^ | **25 ( 3.3)** | **110 ( 1.4)** | **<0.001** |
| Invasive Ventilation ^4^ | 20 ( 2.7) | 264 ( 3.3) | 0.43 |
| Deceased | 2 ( 0.3) | 59 ( 0.7) | 0.22 |

^1^ %=column percentage

^2^ median (Q1-Q3)

^3^ Less-invasive ventilator indications like CPAP or BIPAP machines

^4^ More severe and invasive ventilator indications, including tracheostomy

^5^ Chi-squared test (unless otherwise noted)

^6^ Wilcoxon Rank Sum Test **Supplemental Table C:** Adjusted variable associations with complications (hospitalization, maximum length of hospital stay, moderate ventilation, invasive ventilation) among COVID-19 positive female patients (18-44 years old)

| Variables | Hospitalization | Max LOS  (<1 Day) | Max LOS  (>=1 Day) | Moderate Ventilation | Invasive Ventilation |
| --- | --- | --- | --- | --- | --- |
|  | aOR^1^ (95% CI) | $e^{\hat{\beta}}$^2^ (95% CI) | $e^{\hat{\beta}}$^2^ (95% CI) | aOR^1^ (95% CI) | aOR^1^ (95% CI) |
| Pregnant |  |  |  |  |  |
| No | 1 [Reference] | 1 [Reference] | 1 [Reference] | 1 [Reference] | 1 [Reference] |
| Yes | **10.27 (8.43, 12.54)** | **1.93 (1.74, 2.14)** | **0.80 (0.73, 0.87)** | **2.69 (1.58, 4.43)** | 0.94 (0.55, 1.54) |
| Age (years) [5 years increment]^3^ | **1.11 (1.01, 1.21)** | **1.05 (1.04, 1.07)** | **1.06 (1.03, 1.08)** | 1.07 (0.94, 1.21) | **1.19 (1.08, 1.30)** |
| Race and Ethnicity |  |  |  |  |  |
| Non-Hispanic White | 1 [Reference] | 1 [Reference] | 1 [Reference] | 1 [Reference] | 1 [Reference] |
| Non-Hispanic Black or African American | **0.80 (0.65, 0.98)** | **1.14 (1.07, 1.22)** | 1.04 (0.95, 1.14) | 1.18 (0.68, 2.09) | 0.77 (0.52, 1.14) |
| Non-Hispanic Other | 1.04 (0.85, 1.28) | 0.95 (0.88, 1.02) | **1.12 (1.02, 1.23)** | 1.56 (0.90, 2.75) | 1.23 (0.84, 1.81) |
| Hispanic or Latino | **0.61 (0.51, 0.73)** | **0.90 (0.85, 0.96)** | 0.97 (0.89, 1.05) | 0.93 (0.55, 1.60) | **0.53 (0.36, 0.76)** |
| Insurance |  |  |  |  |  |
| Private | 1 [Reference] | 1 [Reference] | 1 [Reference] | 1 [Reference] | 1 [Reference] |
| Medicaid/Medicare | **1.59 (1.36, 1.87)** | **1.29 (1.22, 1.36)** | **1.14 (1.06, 1.22)** | 1.20 (0.78, 1.86) | 1.13 (0.82, 1.55) |
| Self-Pay | **0.73 (0.58, 0.91)** | **1.07 (1.01, 1.14)** | 0.98 (0.87, 1.10) | 1.32 (0.70, 2.39) | **0.41 (0.21, 0.74)** |
| Other | **1.73 (1.43, 2.09)** | **1.10 (1.03, 1.18)** | **1.17 (1.07, 1.28)** | 1.20 (0.69, 2.05) | **1.55 (1.08, 2.22)** |
| Region |  |  |  |  |  |
| Southeast | 1 [Reference] | 1 [Reference] | 1 [Reference] | 1 [Reference] | 1 [Reference] |
| Northeast | **1.87 (1.55, 2.26)** | **0.74 (0.70, 0.78)** | 1.04 (0.95, 1.13) | 1.09 (0.65, 1.82) | 1.08 (0.72, 1.61) |
| Midwest | **2.18 (1.77, 2.68)** | 1.06 (0.99, 1.13) | 1.07 (0.97, 1.17) | 1.63 (0.94, 2.78) | **1.59 (1.04, 2.42)** |
| West | **2.17 (1.80, 2.62)** | 0.99 (0.94, 1.05) | 1.02 (0.93, 1.11) | 1.43 (0.86, 2.37) | **1.74 (1.20, 2.53)** |
| Missing | **19.44 (14.01, 27.19)** | **1.74 (1.42, 2.13)** | 1.13 (0.99, 1.29) | 1.92 (0.45, 5.70) | **6.23 (3.41, 11.04)** |
| Elixhauser AHRQ weighted Comorbidity Score [10 units incerement]^4^ | **2.04 (1.85, 2.24)** | **1.12 (1.07, 1.17)** | **1.15 (1.11, 1.18)** | **1.30 (1.09, 1.54)** | **1.70 (1.51, 1.92)** |
| Gestational Diabetes |  |  |  |  |  |
| No | 1 [Reference] | 1 [Reference] | 1 [Reference] | 1 [Reference] | 1 [Reference] |
| Yes | **1.75 (1.25, 2.43)** | 0.90 (0.78, 1.03) | 0.99 (0.87, 1.14) | 0.69 (0.25, 1.56) | 0.81 (0.36, 1.61) |
| Decadron and Prednisone |  |  |  |  |  |
| No | 1 [Reference] | 1 [Reference] | 1 [Reference] | 1 [Reference] | 1 [Reference] |
| Yes | **2.68 (2.03, 3.53)** | **1.21 (1.07, 1.37)** | **1.30 (1.18, 1.43)** | **1.83 (1.11, 2.91)** | **2.64 (1.86, 3.70)** |
| Anticoagulant |  |  |  |  |  |
| No | 1 [Reference] | 1 [Reference] | 1 [Reference] | 1 [Reference] | 1 [Reference] |
| Yes | **54.26 (43.73, 67.93)** | **3.09 (2.65, 3.60)** | **1.32 (1.23, 1.41)** | **11.90 (7.91, 18.21)** | **9.79 (7.29, 13.23)** |
| AUC | 0.88 | - | - | 0.85 | 0.89 |
| R^2^ | - | 0.12 | 0.14 | - | - |

^1^ Adjusted odds ratio from mixed-effect logistic regression model (clustering on one-digit zip-code)

^2^ adjusted exponentiated coefficients (mixed-effect exponential regression model clustering on one-digit zip-code) relating to percentage change in expected maximum length of hospital stay

^3^ Change in odds (or change in % of response for exponential model) for each 5 unit increase in predictor

^4^ Change in odds (or change in % of response for exponential model) for each 10 unit increase in predictor

**Supplemental Figures 1A-1E**: Predicted outcomes (hospitalization, maximum length of stay (<1 day, >=1 day), moderate ventilation, and invasive ventilation) vs. Elixhauser AHRQ weighted score, among COVID-19 positive female patients (18-44 years old) by pregnancy status


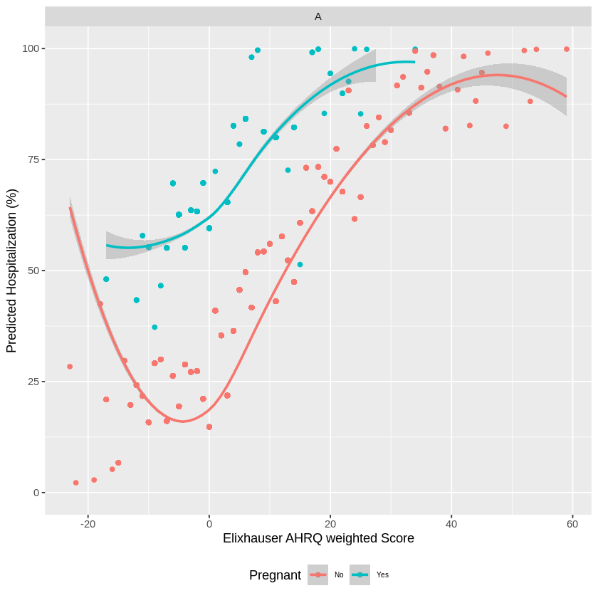

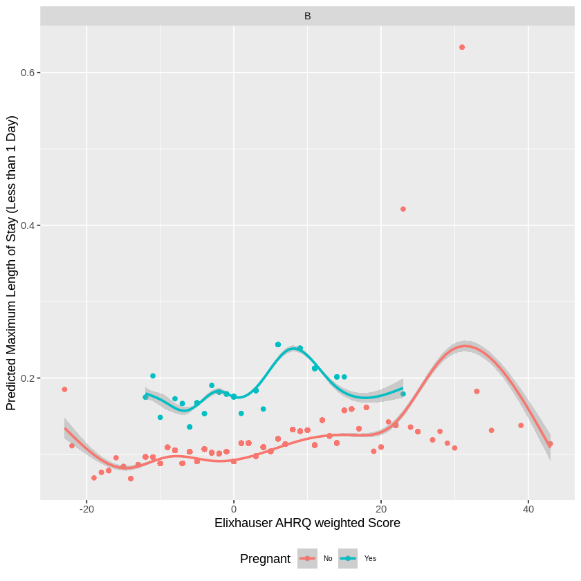

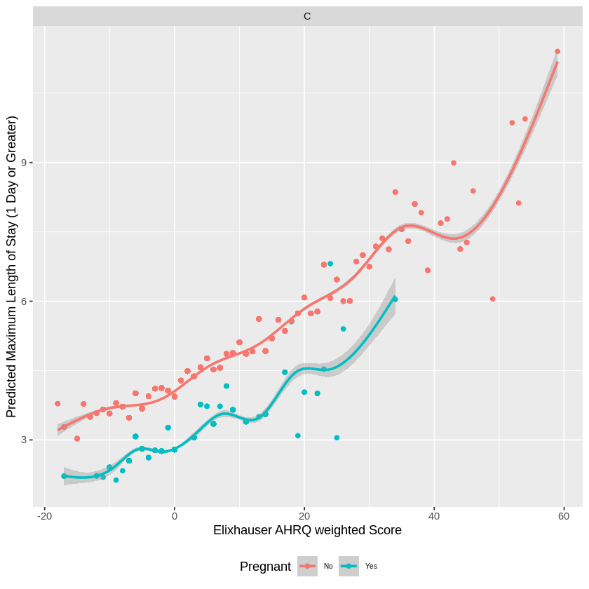

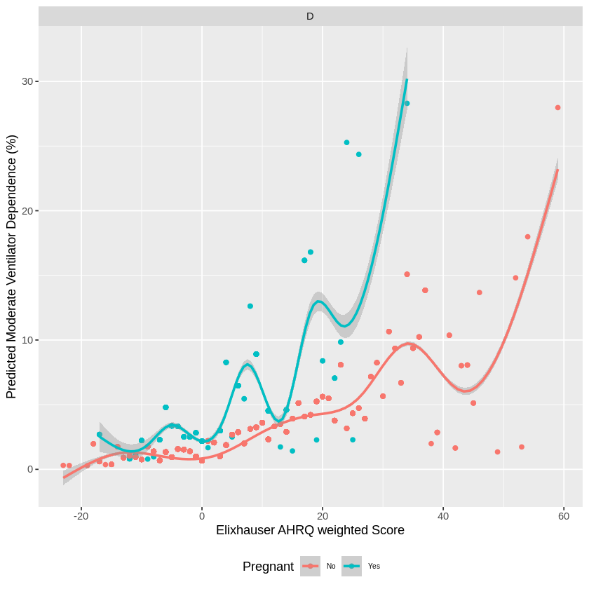

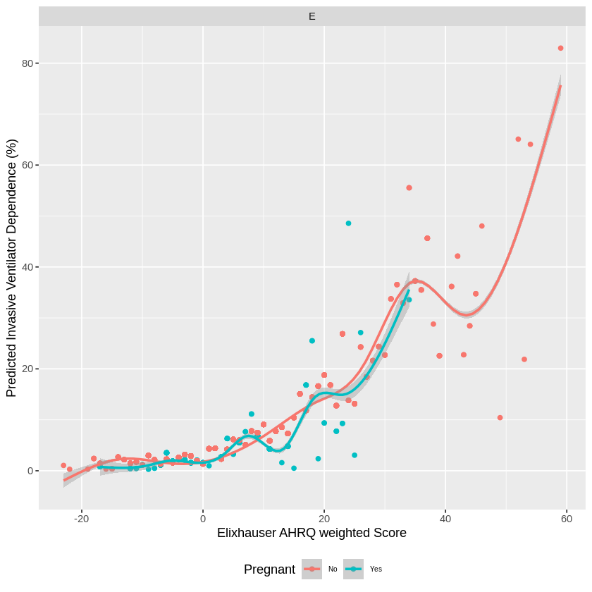


STROBE Statement—Checklist of items that should be included in reports of ***cross-sectional studies***

|  | Item No | Recommendation | Page No |
| --- | --- | --- | --- |
| **Title and abstract** | 1 | (*a*) Indicate the study’s design with a commonly used term in the title or the abstract | Abstract Page |
|  |  | (*b*) Provide in the abstract an informative and balanced summary of what was done and what was found | Abstract Page |
| Introduction | | | |
| Background/rationale | 2 | Explain the scientific background and rationale for the investigation being reported | Manuscript page 1 |
| Objectives | 3 | State specific objectives, including any prespecified hypotheses | Manuscript page 1-2 |
| Methods | | | |
| Study design | 4 | Present key elements of study design early in the paper | Manuscript page 2 |
| Setting | 5 | Describe the setting, locations, and relevant dates, including periods of recruitment, exposure, follow-up, and data collection | Manuscript page 2-3 |
| Participants | 6 | (*a*) Give the eligibility criteria, and the sources and methods of selection of participants | Manuscript page 2-3 |
| Variables | 7 | Clearly define all outcomes, exposures, predictors, potential confounders, and effect modifiers. Give diagnostic criteria, if applicable | Manuscript page 3-5 |
| Data sources/ measurement | 8* | For each variable of interest, give sources of data and details of methods of assessment (measurement). Describe comparability of assessment methods if there is more than one group | Manuscript page 3-5 |
| Bias | 9 | Describe any efforts to address potential sources of bias | Manuscript page 2-3, 13-14 |
| Study size | 10 | Explain how the study size was arrived at | This is a retrospective study which aimed to find all available patients fitting the criteria |
| Quantitative variables | 11 | Explain how quantitative variables were handled in the analyses. If applicable, describe which groupings were chosen and why | Manuscript page 3-6 |
| Statistical methods | 12 | (*a*) Describe all statistical methods, including those used to control for confounding | Manuscript page 5-6 |
|  |  | (*b*) Describe any methods used to examine subgroups and interactions | Manuscript page 6 |
|  |  | (*c*) Explain how missing data were addressed | Manuscript page 5, Tables 1a, 3, 4 |
|  |  | (*d*) If applicable, describe analytical methods taking account of sampling strategy | - |
|  |  | (*e*) Describe any sensitivity analyses | Manuscript page 6 |
| Results | | | |
| Participants | 13* | (a) Report numbers of individuals at each stage of study—eg numbers potentially eligible, examined for eligibility, confirmed eligible, included in the study, completing follow-up, and analysed | Manuscript page 2-3, 7 |
|  |  | (b) Give reasons for non-participation at each stage | Manuscript page 2-3 |
|  |  | (c) Consider use of a flow diagram | - |
| Descriptive data | 14* | (a) Give characteristics of study participants (eg demographic, clinical, social) and information on exposures and potential confounders | Tables 1a, 1b |
|  |  | (b) Indicate number of participants with missing data for each variable of interest | Table 1a |
| Outcome data | 15* | Report numbers of outcome events or summary measures | Table 2 |
| Main results | 16 | (*a*) Give unadjusted estimates and, if applicable, confounder-adjusted estimates and their precision (eg, 95% confidence interval). Make clear which confounders were adjusted for and why they were included | Tables 2-4 |
|  |  | (*b*) Report category boundaries when continuous variables were categorized | Tables 1a, 1b, 4 |
|  |  | (*c*) If relevant, consider translating estimates of relative risk into absolute risk for a meaningful time period | _ |
| Other analyses | 17 | Report other analyses done—eg analyses of subgroups and interactions, and sensitivity analyses | Tables 4, Supplemental Tables C1, C2, D, E |
| Discussion | | | |
| Key results | 18 | Summarise key results with reference to study objectives | Manuscript page 9-10 |
| Limitations | 19 | Discuss limitations of the study, taking into account sources of potential bias or imprecision. Discuss both direction and magnitude of any potential bias | Manuscript page 13-14 |
| Interpretation | 20 | Give a cautious overall interpretation of results considering objectives, limitations, multiplicity of analyses, results from similar studies, and other relevant evidence | Manuscript page 10-13 |
| Generalisability | 21 | Discuss the generalisability (external validity) of the study results | Manuscript page 10, 13-14 |
| Other information | | | |
| Funding | 22 | Give the source of funding and the role of the funders for the present study and, if applicable, for the original study on which the present article is based | Manuscript page 15 |

*Give information separately for exposed and unexposed groups.

**Note:** An Explanation and Elaboration article discusses each checklist item and gives methodological background and published examples of transparent reporting. The STROBE checklist is best used in conjunction with this article (freely available on the Web sites of PLoS Medicine at http://www.plosmedicine.org/, Annals of Internal Medicine at http://www.annals.org/, and Epidemiology at http://www.epidem.com/). Information on the STROBE Initiative is available at www.strobe-statement.org.
